# New Insights into Polygenic Score–Lifestyle Interactions for Cardiometabolic Risk Factors from Genome-Wide Interaction Analyses

**DOI:** 10.3390/nu15224815

**Published:** 2023-11-17

**Authors:** Shannon D’Urso, Liang-Dar Hwang

**Affiliations:** Institute for Molecular Bioscience, The University of Queensland, Brisbane, QLD 4067, Australia; s.durso@uq.edu.au

**Keywords:** gene–environment interaction, polygenic score, lifestyle, BMI, blood lipid, cardiometabolic risk, diet, physical activity, polygenic risk score, blood pressure

## Abstract

The relationship between lifestyles and cardiometabolic outcomes varies between individuals. In 382,275 UK Biobank Europeans, we investigate how lifestyles interact with polygenic scores (PGS) of cardiometabolic risk factors. We identify six interactions (PGS for body mass index with meat diet, physical activity, sedentary behaviour and insomnia; PGS for high-density lipoprotein cholesterol with sedentary behaviour; PGS for triglycerides with meat diet) in multivariable linear regression models including an interaction term and show stronger associations between lifestyles and cardiometabolic risk factors among individuals with high PGSs than those with low PGSs. Genome-wide interaction analyses pinpoint three genetic variants (*FTO* rs72805613 for BMI; *CETP* rs56228609 for high-density lipoprotein cholesterol; *TRIB2* rs4336630 for triglycerides; *P_Interaction_* < 5 × 10^−8^). The associations between lifestyles and cardiometabolic risk factors differ between individuals grouped by the genotype of these variants, with the degree of differences being similar to that between individuals with high and low values for the corresponding PGSs. This study demonstrates that associations between lifestyles and cardiometabolic risk factors can differ between individuals based upon their genetic profiles. It further suggests that genetic variants with interaction effects contribute more to such differences compared to those without interaction effects, which has potential implications for developing PGSs for personalised intervention.

## 1. Introduction

Genome-wide association studies (GWAS) have revealed that the genetic architecture of human complex traits is mostly highly polygenic, typically due to the action of thousands of genetic variants of small effect [1]. Polygenic scores (PGSs) or polygenic risk scores (PRSs) are the summed effects of the alleles carried by an individual that can be used to predict an individual’s phenotype [2,3]. PGSs have provided informative predictions for highly heritable traits such as height and schizophrenia, with proportions of variance explained (*R*^2^ or Nagelkerke *R*^2^) as being 24.4% and 18%, respectively [4,5], and these PGSs have been integrated into risk prediction models, along with traditional clinical, biochemical, lifestyle, and historical risk factors, to improve the risk prediction of cardiovascular disease and diabetes [6,7,8,9].

**Figure 1:**
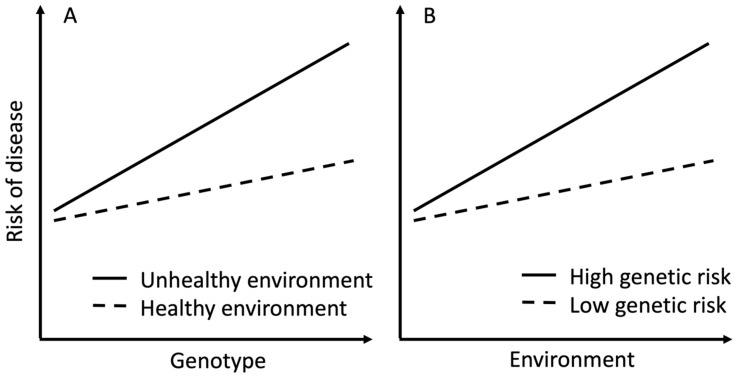
An example of gene–environment interaction. (**A**) The association between genotype and risk of disease varies between environmental exposures. (**B**) The association between environmental exposure and risk of disease varies according to genetic risk.

There is growing interest in understanding whether PGSs interact with environmental factors to affect health risks. Statistically speaking, a gene–environment interaction can be conceptualised as either an effect of a genotype on a trait or disease risk differing between individuals with different levels of environmental exposure (Figure 1A) or an effect of an environmental exposure on a trait or disease risk differing between individuals with different genotypes (Figure 1B). Several biological models have been proposed to interpret a gene–environment interaction, such as a genotype increasing a person’s exposure to an environmental factor or a genotype mediating the effect of exposure on a disease [10]. Studies using data from large genetic studies such as the UK Biobank have shown PGS–lifestyle interactions for body mass index (BMI) [11,12], cardiovascular disease [13], type 2 diabetes [13], and lipid levels [14], but not for the incidence of dementia [15]. Other studies have found no evidence for PGS–lifestyle interactions on cardiovascular diseases [16] or cancer [17]. A PGS–environment interaction is often interpreted from the point of risk prediction, for example, that genetic contribution to disease risk depends on the level of environmental risk factors or, in the case of PGS–lifestyle interaction, obesogenic environment accentuates the risk in genetically susceptible individuals.

Given that chronic conditions can often be prevented, ameliorated, and even treated through behavioural change interventions, we are interested in whether a PGS-lifestyle interaction can be applied to “personalised intervention” to inform on which lifestyle interventions are more effective than others based on an individual’s genetic risk. This goal can be achieved if one could show that the effects of lifestyles on an outcome of interest are different across PGS strata, e.g., the effect of exercise on reducing BMI is higher in individuals with a higher genetic risk than in those with a lower genetic risk. As a starting point, here we investigate the associations between lifestyle behaviours (including diet, physical activity, and sleep) and cardiometabolic risk factors (including BMI, lipid levels, and blood pressure) across strata of PGS, created using the published GWAS of cardiometabolic risk factors, in up to 382,375 UK Biobank participants. Then, we explore individual genetic variants that may contribute to the differential effects of lifestyles on cardiometabolic risk factors across different PGS strata. Based on our findings, in our discussion, we propose a new PGS that may have future implications for personalised intervention.

## 2. Method

### 2.1. Sample and Genotyping

The UK Biobank is a prospective cohort study consisting of over 500,000 participants (aged 37–73 years old; 54.4% female; 5% of those invited) from 21 centres across England, Wales, and Scotland with the goal of improving the prevention, diagnosis, and treatment of diseases of middle and old age [18]. Participants responded to questionnaires to provide information on health and lifestyle in a baseline survey, took part in clinical assessments, and provided blood, urine, and saliva samples for biomarker and genetic assays.

All UK Biobank participants have been genotyped using the Affymetrix UK BiLEVE Axiom array or Affymetrix UK Biobank Axiom array comprising 805,426 markers in the official release. Genotype imputation was performed using IMPUTE2 software and UK10K haplotype and Haplotype Reference Consortium reference panels [18]. Single nucleotide polymorphisms (SNPs) with a call rate < 90%, minor allele frequency < 0.005, imputation score < 0.3, and Hardy–Weinberg equilibrium score of *p* < 1.0 × 10^−6^ were excluded, with a total of 11,183,892 SNPs remaining in the analyses.

This study only included unrelated individuals of European ancestry (N up to 382,375; 54% females, aged between 38 and 72 years old, with a median age of 58 and a mean age of 56 years) to avoid bias due to population stratification. Related individuals (i.e., one from each pair of third-degree relatives or closer) were excluded based on their kinship coefficients estimated using the software KING (https://www.kingrelatedness.com/, accessed on 10 October 2023) [19]. Participants’ ancestries were determined by generating genetic principal components values (PCs) in the 1000 Genome samples, followed by a K-means clustering analysis using the first four PCs. Those clustering with the European cluster were classified as having European ancestry. We excluded individuals who did not report their ethnic background as one of “British”, “Irish”, “White”, or “Any other white background”. We also excluded individuals with aneuploidy (*n* = 652), poor quality genotypes (outliers in heterozygosity or missingness rate; *n* = 968), and a mismatch between reported and inferred gender (*n* = 378), as identified by the UKBB, as well as those who had withdrawn from the UK Biobank as of February 2020. Participants with missing data were excluded from the corresponding analyses.

### 2.2. Ethics Statement

The UK Biobank study was approved by the UK National Health Service’s National Research Ethics Service. Written consent was obtained from both the participants and their parents (for subjects younger than 18 years old).

### 2.3. Lifestyle Behavioural Factors

Six lifestyle behaviours were extracted or derived from the UK Biobank. Two diet scores were derived following a recent GWAS of dietary intake [20]. In brief, principal component analyses were performed on self-reported food intake measures from the generic diet questionnaire. The first two principal components represented a diet consisting largely of meat (high weights from the intake of processed meat, poultry, beef, lamb and pork; referred to henceforth as “meat diet”) and a diet comprising largely of fish and plant-based food (high weights from the intake of raw and cooked vegetables, fruit, and oily and non-oily fish; referred to as the “plant-fish diet”).

We extracted two physical activity scores—the total metabolic equivalent task (MET) minutes of exercise per week (based on the International Physical Activity Questionnaire; referred to as “physical activity”) and the time spent on sedentary activities (hours/week; referred to as “sedentary time”). MET values were pre-calculated using the time spent undertaking walking, moderate physical activity, and vigorous physical activity multiplied by the MET values corresponding to the energy cost of each physical activity, i.e., 2.5 for slow walking, 3.3 for moderate walking, 5 for fast walking, 4 for moderate exercise, and 8 for vigorous exercise (UK Biobank Field 22040). Time spent on sedentary activities per week was calculated by summing the number of hours spent driving, using a computer, and watching television per week (UK Biobank Fields 1070, 1080, 1090).

We extracted two sleep-related traits: sleep duration and insomnia (UK Biobank Fields 1160, 1200). Sleep duration was based on participants’ answers to the question “About how many hours sleep do you get in every 24 h? (please include naps)”. Insomnia was based on the answer to the question “Do you have trouble falling asleep at night or do you wake up in the middle of the night?”, and the answers were “Never/Rarely”, “Sometimes”, and “Usually” (coded as 1, 2, and 3, respectively, in the analysis).

### 2.4. Cardiometabolic Risk Factors

Six cardiometabolic risk factors were extracted from the UK Biobank. BMI was constructed from height and weight (UK Biobank Field 20001). Plasma concentrations of high-density lipoprotein cholesterol (HDL-C), low-density lipoprotein cholesterol (LDL-C), and triglycerides (UK Biobank Fields 30760, 30780, 30870) were measured via enzymatic protective selection analysis on a Beckman Coulter AU5800 (Beckman Coulter Ltd., High Wycombe, UK). Blood pressure was measured using the Omron 705 IT electronic blood pressure monitor (OMRON Healthcare Europe B.V., Hoofddorp, The Netherlands). Systolic blood pressure (SBP) and diastolic blood pressure (DBP) were derived as the mean of the two recorded automated measurements (UK Biobank Fields 4079, 4080). These six cardiometabolic risk factors are metabolic syndromes that have been used to diagnose cardiometabolic disorders [21]. We used data obtained during the initial Assessment Centre visit to ensure all data were collected at the same time-point.

### 2.5. Polygenic Scores

Weighted PGSs were constructed for the six cardiometabolic risk factors using the following formula:

$$PGS=\sum_{i=1}^{n}\beta_{i}\times {SNP}_{i}$$

where SNP is the number of trait-increasing alleles (0, 1, or 2), and β is the effect size from the association between a SNP and the cardiometabolic risk factor from a published GWAS. Each PGS was calculated as the sum of the number of trait-increasing alleles multiplied by the β for all independent genome-wide significant SNPs for the respective trait.

We used SNPs identified in GWAS of Europeans that did not include UK Biobank participants. These included 97 SNPs for BMI identified by the GIANT consortium [22]; 96, 82, and 60 SNPs for serum concentrations of HDL-C, LDL-C, and triglycerides identified by the Global Lipid Genetic Consortium [23]; and 68 and 71 SNPs for SBP and DBP identified by the International Consortium of Blood Pressure [24,25,26,27] (see Supplementary Table S1 for a full list of SNPs).

### 2.6. Statistical Analysis

Individuals were grouped by PGS quartiles, where individuals in the bottom 25% (Q1) and the top 25% (Q4) of the PGS quartile groups were defined as having a low and a high genetic risk, respectively. Likewise, individuals were grouped based on the levels of their lifestyle factors, with those in the bottom and top tertile for each lifestyle (i.e., bottom third and top third) being defined as having a low level and high level of that lifestyle, respectively. We used tertile because insomnia was coded into three levels in the UK Biobank. The lifestyle behavioural factors (meat diet, plant–fish diet, physical activity, sedentary time, sleep duration, and insomnia) and cardiometabolic risk factors (BMI, HDL-C, triglycerides, SBP, and DBP) were transformed into z-scores prior to statistical analyses. As the lifestyle behavioural factors, as well as cardiometabolic risk factors, were correlated with each other, we conducted principal components analyses to estimate the approximate number of independent variables. Given that 5 PCs and 2 PCs accumulatively accounted for more than 90% of the variance in the 6 lifestyles and 6 cardiometabolic risk factors respectively, a Bonferroni-corrected significance threshold of *p* = 0.05/(2 × 5) = 0.005 was used.

A multivariable linear regression model including an interaction term (i.e., Lifestyle × PGS interaction analysis) was constructed to assess the interaction between each lifestyle factor and the PGS for the corresponding cardiometabolic risk factor (Formula (1)). Covariates included age, sex, genotype array, and the first 10 genetic PCs. Following previous recommendations to control for possible confounding effects of the PGS and covariates [28], additional interaction terms for PGS with non-heritable covariates were also included in the model.

(1) $$\begin{array}{cc} Cardiometabolic & risk\;factor\;\sim \;Lifestyle+PGS+Lifestyle\times PGS+Age+Sex+Array+PCs+PGS\times Age\\ & +PGS\times Sex+PGS\times Array \end{array}$$

A second multivariable linear regression model was constructed to assess the association between lifestyles and cardiometabolic risk factors in PGS-stratified groups (Formula (2)). This analysis is hereafter referred to as the PGS-stratified association analysis.

(2) Cardiometabolic risk factor ~ Lifestyle+Age+Sex+Array+PCs

Given that the distributions of the cardiometabolic risk factors were skewed (Supplementary Figure S1), there was a possibility of heteroskedasticity, where the variances of cardiometabolic risk factors differ across the different PGS groups, and this would violate the homoscedasticity assumption of linear regression. We, therefore, performed sensitivity analyses using inverse-normal transformed cardiometabolic risk factors.

To investigate the potential contribution of individual genetic variants to the interactions, we conducted genome-wide interaction analyses (Formula (3)) for lifestyle–cardiometabolic risk factor combinations that were significant in the PGS–lifestyle interaction analyses.

(3) Cardiometabolic risk factor ~ lifestyle+SNP+lifestyle×SNP+Age+Sex+Batch+PCs

We used R version 3.5.1 for all linear regression analyses and PLINK 1.90 [29] for genome-wide interaction analysis.

## 3. Results

**Figure 2:**
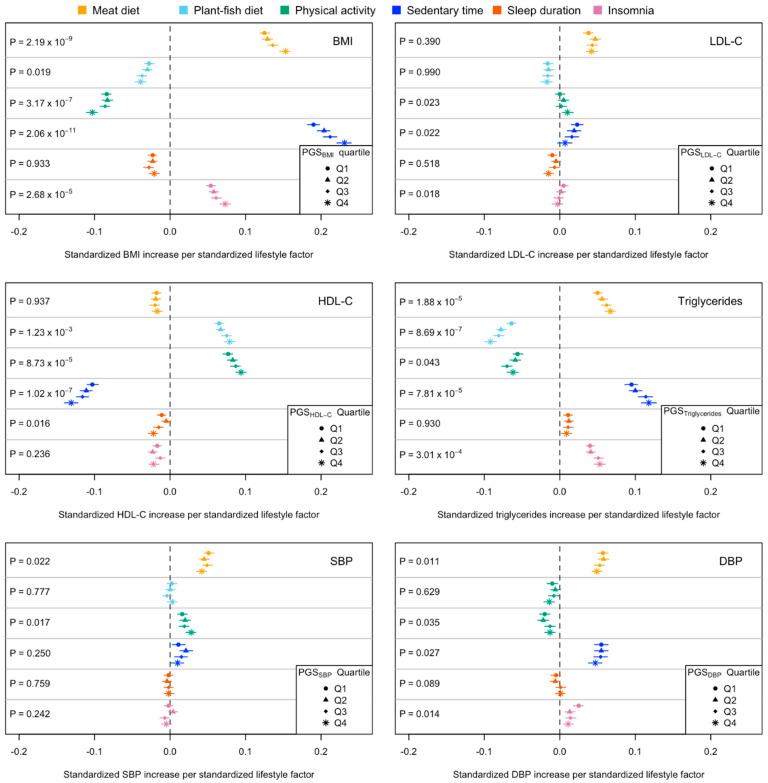
Effects (β and 95% confidence intervals) of lifestyle behavioural factors on cardiometabolic risk factors among UK Biobank individuals of European ancestry across polygenic scores strata. Q1 and Q4 represent the groups with low (bottom 25%) and high (top 25%) genetic risk, respectively. β and 95% confidence intervals were obtained from the PGS-stratified association analyses. *p* values were obtained from the PGS–lifestyle interaction analyses using the whole sample.

**Figure 3:**
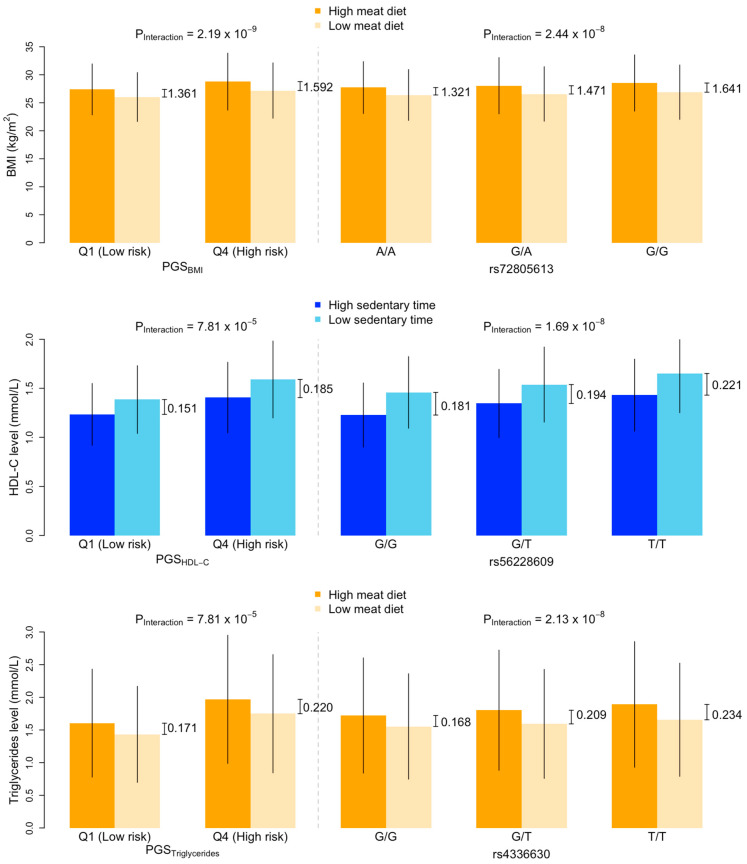
Comparison of cardiometabolic risk factors by levels of lifestyles between PGS groups and genotype groups. Top panel shows the means and standard errors of body mass index (BMI) by levels of meat diet and (i) low and high PGS_BMI_ (**left**) and (ii) rs72805613 genotype (**right**). *p* values are the interaction terms between the PGS and meat diet (**left**) and the rs7285613 genotype and meat diet (**right**) on BMI obtained via a multivariable linear regression model. High-meat diet and low-meat diet are defined based on the top and bottom tertiles of meat intake scores. (i.e., bottom third and top third of meat intake scores). Differences in mean BMI between high- and low-meat diet are labelled for each of the PGS_BMI_ and genotype groups. Middle panel shows the means and standard errors of serum concentrations of high-density lipid cholesterol (HDL-C) by levels of sedentary time and (i) low and high PGS_HDL-C_ (**left**) and (ii) rs56228609 genotype (**right**)**.** Bottom panel shows the means and standard errors of serum concentrations of triglycerides by levels of meat diet and (i) low and high PGS_Triglycerides_ (**left**) and (ii) rs4336630 genotype (**right**).

Descriptive statistics of the cardiometabolic risk factors grouped by their PGSs are summarised in Supplementary Table S2. In the PGS–lifestyle interaction analyses, we found four, three, and four significant interactions for BMI, HDL-C, and triglycerides (Figure 2; Supplementary Table S3), among which four, one, and one interactions, respectively, were significant in the sensitivity analyses (*p* < 0.05; Supplementary Table S4), including PGS_BMI_ with meat diet, physical activity, sedentary time, PGS_HDL-C_ with sedentary time, and PGS_Triglycerides_ with meat diet. In the PGS-stratified association analyses, the absolute effects of lifestyles on cardiometabolic risk factors tended to be larger in the high PGS groups compared to those in the low PGS groups. For example, the effect of meat diet on BMI was larger among individuals in the high PGS_BMI_ group (β [95% confidence intervals] = 0.152 [0.145, 0.159]) than that in the low PGS_BMI_ group (β = 0.125 [0.119, 0.132]); the negative effect of sedentary time on HDL-C was larger in the high PGS_HDL-C_ group (β = −0.131 [−0.140, −0.122]) than that in the low PGS_HDL-C_ group (β = −0.103 [−0.110, −0.095]). A similar pattern was observed when comparing the mean of cardiometabolic risk factors between those with high and low levels of lifestyle factors. For example, in the high PGS_BMI_ group, the difference in BMI between those with a high- and low-meat diet was 1.592 kg/m^2^, whereas in the low PGS_BMI_ group, the difference in BMI between those with a high- and low-meat diet was only 1.361 kg/m^2^ (top panel in Figure 3, Supplementary Table S5).

### Genome-Wide Interaction Analysis

**Figure 4:**
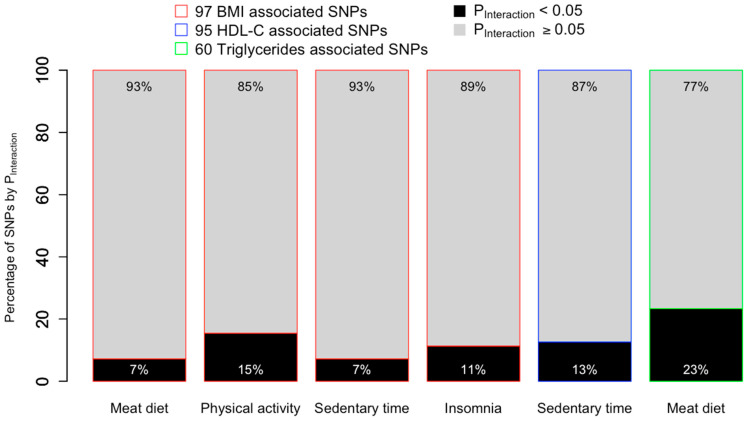
Percentage of cardiometabolic risk factor-associated SNPs that interact with lifestyle factors to affect their corresponding cardiometabolic outcomes in the SNP-based interaction analyses. Values within the black portion of the bar chart represent the percentage of GWAS-identified SNPs that were significant in the SNP-based interaction analyses (P_Interaction_ < 0.05), whereas values within the grey portion of the bar represent the percentage of GWAS identified SNPs with P_Interaction_ ≥ 0.05. For example, among the 97 BMI-associated SNPs, 7 SNPs (equivalent of 7%) interacted with meat diet with P_Interaction_ < 0.05.

We conducted genome-wide interaction analyses for the six lifestyle–cardiometabolic risk factor combinations identified via the PGS–lifestyle interaction analyses. We first examined the SNPs used to construct PGSs, i.e., SNPs identified in the original main effects GWAS with *p* < 5 × 10^−8^, to assess which SNPs contributed to the PGS–lifestyle interactions. Between 7% and 23% of cardiometabolic risk factors associated SNPs interacted with lifestyle factors (*p* < 0.05) (Figure 4). Four BMI associated SNPs interacted with more than one lifestyle; for example, rs1558902 interacted with meat diet, physical activity, and sedentary time (see Supplementary Tables S6–S8 for full results).

**Figure 5:**
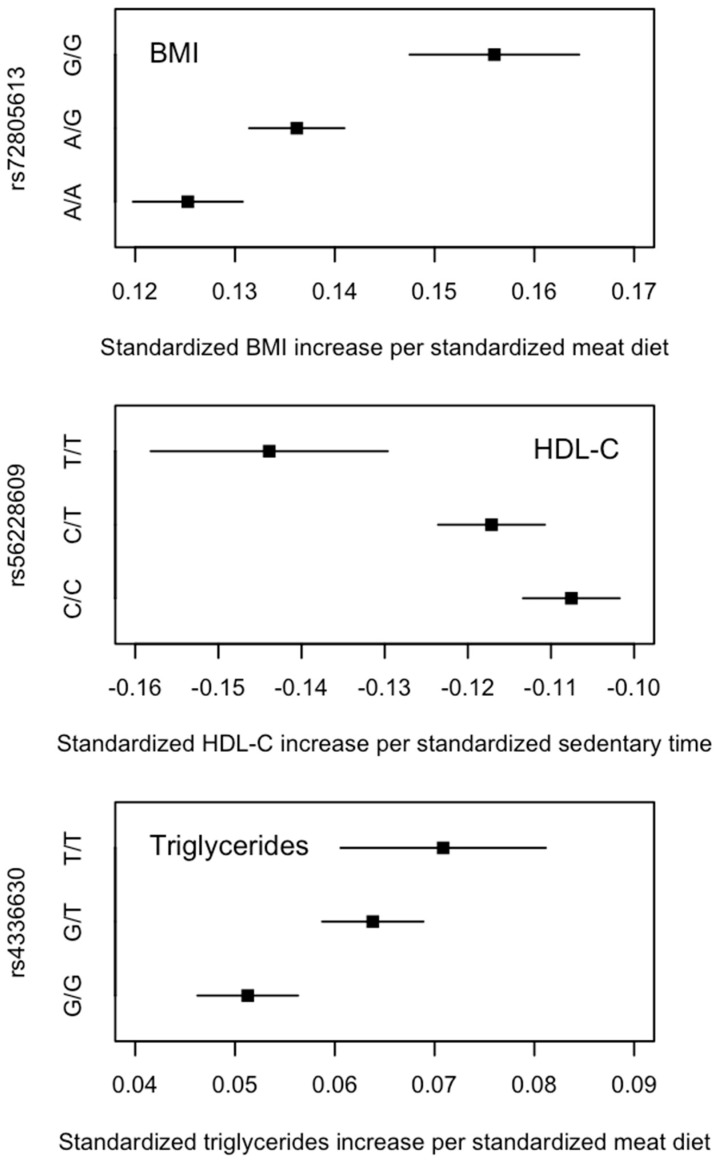
Effects (β and 95% confidence intervals) of lifestyle behavioural factors on cardiometabolic risk factors among all UK Biobank individuals of European ancestry by genotypes of SNPs that were significant in the genome-wide interaction analyses. β and 95% confidence intervals were obtained from the genotype-stratified association analyses.

When examining the full set of SNPs, three SNP–lifestyle interactions reached the uncorrected genome-wide significance threshold of *p* < 5 × 10^−8^ (Supplementary Table S9), all of which (or SNPs in linkage disequilibrium) had been associated with their corresponding risk factors (as main effects). When grouping the individuals by their genotypes at these loci, there was a clear pattern indicating that the associations between lifestyles and cardiometabolic risk factors were different between genotype groups; for example, the effect of meat diet on BMI increased with the number of rs72805613 G alleles (Figure 5). The difference in the effects between those with no G allele and those with two G alleles was 0.031 (i.e., β = 0.125 and 0.156 in the A/A and G/G groups, respectively) (Supplementary Table S10), similar to the difference in the effects between those with high and low PGS_BMI_. (i.e., β = 0.125 and 0.152 in the low and high PGS_BMI_ groups, respectively (Supplementary Table S3). Furthermore, among individuals with no G allele, the difference in BMI between those with a high- and low-meat diet was 1.321 kg/m^2^, whereas among those with two G alleles, the difference in BMI between those with a high- and low-meat diet was 1.641 kg/m^2^, similar to the differences in the high and low PGS_BMI_ groups (top panel in Figure 3). A similar pattern was also observed for the comparisons between rs56228609 genotypes and PGS_HDL-C_ and between rs4336630 and PGS_Triglycerides_ (Figure 3).

## 4. Discussion

In this study, we demonstrated that the associations between lifestyles and cardiometabolic risk factors can differ between individuals with high and low PGSs for the corresponding cardiometabolic risk factors, and we identified six PGS–lifestyle interactions for cardiometabolic risk factors, including four for BMI, one for HDL-C, and one for triglycerides. Genome-wide interaction analyses revealed that most of the SNPs used to construct traditional PGSs (from the main effects GWAS) did not individually interact with lifestyles and pinpointed specific genetic variants that may explain the differential effects of lifestyle modification on cardiometabolic risk factors between individuals.

Consistent with findings from previous studies [11,12,30,31], we found a positive interaction between obesogenic lifestyles (i.e., high-meat diet, low physical activity level, more time spent on sedentary activities, and poor-quality sleep) and PGS_BMI_. In contrast to previous studies interpreting these interactions from the point of view of risk prediction, as lifestyle factors may modify the effect of PGS_BMI_ on BMI, here we viewed them from the point of view of risk intervention and showed that the PGS_BMI_ may modify the associations between lifestyle factors and BMI, suggesting that individuals with a high genetic risk may be more amendable to lifestyle interventions. The difference in the associations between PGS_BMI_ groups could be due to individuals with different genetic profiles responding to environmental exposures differently. For example, the association between meat diet and BMI was stronger among carriers of the *FTO* rs1558902 A allele than those with the T allele (Supplementary Table S5). Associations with sleep duration did not differ between PGS_BMI_ groups. This could be due to the U-shaped relationship between sleep duration and obesity [32], with both short sleepers and long sleepers having an increased risk of obesity [33]. We are aware of that association does not necessarily imply causation, and even if there was a causal relationship, the direction of the effect requires further investigations, i.e., while living an obesogenic lifestyle can lead to a higher BMI, having a high BMI may also drive people to change their lifestyles.

We found evidence for interactions between sedentary time and PGS_HDL-C_ and between meat diet and PGS_Triglycerides_. This replicated the result from a recent study, also using data from the UK Biobank, that found a PGS–lifestyle interaction for triglycerides but not for total cholesterol or LDL-C [14]. The study constructed an overall healthy lifestyle score based on multiple factors, including smoking and BMI, in addition to diet and physical activity, whereas we examined each of the lifestyles separately. Nevertheless, results from both studies suggest that individuals with high PGSs may benefit more from adherence to a healthy lifestyle.

We observed weak interactions for PGS_SBP_ (with meat intake and physical activity) and PGS_DBP_ (with all lifestyles except for sleep duration), but none were significant when considering multiple testing. Interestingly, there is a trend that associations between lifestyles and blood pressures were larger among individuals with high PGSs than those with low PGSs, which is the opposite of the trend observed for BMI and lipid levels. This warrants further investigations using PGS creased using SNPs identified in larger GWASs, such as a more recent work conducted by the International Congress for Blood Pressure [34].

Among the 11 interactions identified in the main analyses, 5 did not pass sensitivity analyses that used inverse-normal transformed data. This suggests the presence of heteroskedasticity where the variance of outcome variables (i.e., cardiometabolic risk factors) differs across levels of predicting variables (i.e., PGS groups). As shown in Supplementary Table S2, the variance in the cardiometabolic risk factors tends to increase from low PGS group to the high PGS group. Since homoscedasticity is one of key assumptions of the linear regression model, investigations of interactions using heteroskedastic data may lead to biased estimates. This highlights the importance of including sensitivity analyses using either inverse-normal transformed data to reduce the influence of extreme values and make the residuals approximately homoscedastic or using other methods that do not assume homoscedasticity of the data.

The majority of the individual SNPs identified in previous main effects GWAS did not interact with lifestyles in the present study. If not due to lack of statistical power, this suggests that there could be different biological pathways underlying disease aetiology and disease intervention; that SNPs contributing to the development of a trait or disease do not necessarily inform the extent to which carriers of specific alleles respond to an intervention. Additionally, there could be a distinct pathway for each lifestyle–disease outcome combination. Taking the BMI associated SNPs as an example, among the seven SNPs showing an interaction, four interacted with more than one lifestyle, whereas the remaining three only interacted with one lifestyle in this study.

Our genome-wide interaction analyses pinpointed three SNPs within the *FTO*, *CETP*, and *TRIB2* genes that may be the main drivers of the PGS–lifestyle interactions for BMI, HDL-C, and triglycerides, respectively. The *FTO* rs72805613 SNP is in high linkage disequilibrium with the widely studied *FTO* rs1558902 SNP (r^2^ = 0.841). A previous 2-year intervention study of 742 obese adults showed that individuals with the *FTO* rs1558902 A allele benefitted more from a high-protein diet for weight loss and the improvement of body composition and fat distribution [35], which is concordant with our finding showing that the increase in BMI per unit increase in meat diet score was larger among individuals with the rs72805613 G allele. However, a later meta-analysis of eight randomised controlled trials suggested that the response to intervention of diet, physical activity, or a drug-based approach did not differ according to an individual’s *FTO* genotype [36]. *CETP* encodes a protein involved in the transfer of cholesteryl ester from HDL-C to other lipoproteins, and the SNPs within the *CETP* gene have been associated with serum HDL-C concentration across multiple ethnic groups [37,38]. *TRIB1* encodes a tribbles pseudokinase that has been found to be involved in lipid metabolism [39], and variation within this gene has been associated with serum triglycerides concentrations in Europeans [23]. Future studies are required to replicate these results and understand how genetic variation in these genes underlies differential associations between lifestyles and cardiometabolic risk factors.

Here we propose a new polygenic score—a polygenic interaction score (PGIS)—that is constructed using SNPs with an interaction effect identified in a genome-wide interaction analysis rather than SNPs identified in the main effects GWAS. We hypothesize that by including only SNPs that are involved in the interaction to construct PGIS, (i) a PGIS–environment interaction will be much stronger than a PGS–environment interaction, and (ii) the association between an environmental exposure (e.g., physical activity levels) and an outcome of interest (e.g., BMI) will differ more greatly between low PGIS and high PGIS groups compared to that between high PGS and low PGS groups. We have shown that by simply grouping individuals by the genotype of one SNP rs72805613 identified in the genome-wide interaction analysis, the difference in the associations between meat intake and BMI between genotype groups is similar to the differences in the associations between PGS_BMI_ groups. We propose that PGIS could, therefore, help to prioritize or shortlist intervention items by informing which intervention or treatment may have a larger effect, as well as which may be less effective based upon an individual’s genetic profile.

While risk management recommendations are often given in a one-size-fits-all approach, the results of this study suggest that, depending on the type of risk factors, certain lifestyle interventions may provide a greater benefit to individuals with a strong genetic predisposition to that cardiometabolic risk factor. This could partly be due to an individual’s genetically predisposed body system responding to environmental factors in a unique way. Similarly, our results suggest that certain interventions may be more effective than others in reducing cardiometabolic risk, which could be used to inform public health interventions, such as the allocation of resources in a more case-by-case manner when genotype data are available. This may also have additional flow-on benefits, as there is evidence to suggest that individuals are more motivated to adhere to interventions if medical advice is driven by genetic information [40]. Nevertheless, we note that our results need to be replicated in different study designs, such as randomised controlled trials.

Our results are based on individuals in the UK Biobank, with the majority being females, middle class, and middle-aged; therefore, the results may not generalize to other populations. As the difference in associations between PGS groups was small, our findings, by themselves, may have limited clinical impacts. Given that we defined the high- and low-risk groups using the top and bottom 25% of PGS for all cardiometabolic risk factors, further investigation is required to identify clinically meaningful cut-offs, which could be disease-specific. More importantly, an association can be driven by confounding, biases or reverse causation and hence is not equal to causation. Whether the difference in the associations between lifestyles and cardiometabolic risk factors between high- and low-PGS groups indicates differences in the effects of an intervention requires further investigation in randomised controlled trials. Finally, a major challenge involved in constructing our proposed PGIS is that much larger sample sizes are required to detect interaction effects than main genetic effects, and the exposure and outcome need to be available for the same individuals. This may be achievable in the near future with the global increase in the number of large biobanks [41].

## 5. Conclusions

We showed that the associations between lifestyles and cardiometabolic risk factors can differ according to genetic predisposition to the respective cardiometabolic risk factors. We also showed that the difference in the associations between high and low PGS groups was small, and this could be partly due to most of the SNPs used to construct PGSs not being involved in the interaction. This finding highlights the importance of identifying genetic variants involved in interactions and provides a new research direction for the development of PGS for personalised intervention.

## Data Availability

Human genotype and phenotype data on which the results of this study were based were accessed via the UK Biobank (http://www.ukbiobank.ac.uk/ (accessed on 27 June 2019)) with accession ID 53641. The genotype and phenotype data are available upon application from the UK Biobank (http://www.ukbiobank.ac.uk/). The datasets generated during and/or analysed during the current study are available from the corresponding author upon reasonable request.

## Supplementary Materials

The following supporting information can be downloaded at: https://www.mdpi.com/article/10.3390/nu15224815/s1, Figure S1: Histograms showing the distribution of cardiometabolic risk factors among 382,275 unrelated individuals of Europeans in the UK Biobank; Table S1: SNP associated with cardiometabolic risk factors used to construct polygenic scores; Table S2: Descriptive statistics of cardiometabolic risk factors and lifestyle factors grouped by the polygenic risks of cardiometabolic risk factors in the UK Biobank participants of European ancestry; Table S3: Association between lifestyles and cardiometabolic risk factors in polygenic scores (PGS) stratified groups and the interaction between PGS and lifestyles in the UK Biobank participants of European ancestry; Table S4: Sensitivity analysis of the association between lifestyles and inverse-normal transformed cardiometabolic risk factors in polygenic scores (PGS) stratified groups and the interaction between PGS and lifestyles in the UK Biobank participants of European ancestry; Table S5: Descriptive statistics of cardiometabolic risk factors in the UK Biobank participants of European ancestry grouped by tertiles of each lifestyle factor; Table S6: SNP by lifestyle interaction for body mass index (BMI) in the UK Biobank participants of European ancestry; Table S7: SNP by lifestyle interaction for high-density lipid cholesterol (HDL-C) in the UK Biobank participants of European ancestry; Table S8: SNP by lifestyle interaction for triglycerides in the UK Biobank participants of European ancestry; Table S9: Top SNPs interacting with lifestyles to affect BMI, HDL-C and triglycerides; Table S10: Descriptive statistics of cardiometabolic risk factors in the UK Biobank participants of European ancestry by the levels (tertile) of each lifestyle factor.
