# Supplementary material for: New Insights into Polygenic Score–Lifestyle Interactions for Cardiometabolic Risk Factors from Genome-Wide Interaction Analyses
Source: Nutrients. 2023 Nov 17;15(22):4815. doi: 10.3390/nu15224815 (PMC10675788; doi:10.3390/nu15224815)
Supplement: Supplementary file 1 [file nutrients-15-04815-s001.zip › Supplementary Figure S1.pdf]

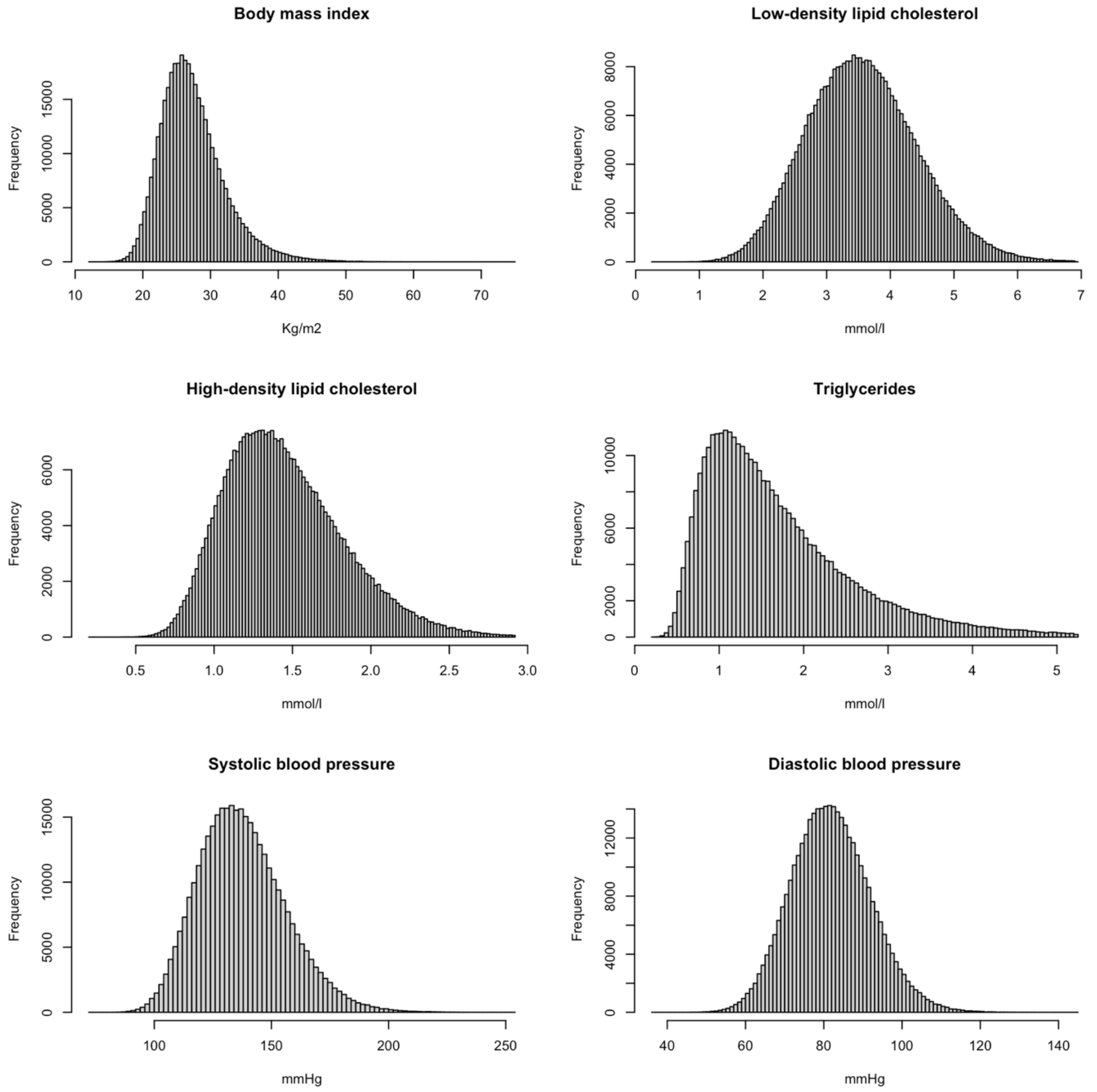

Supplementary Figure S1. Histograms showing the distribution of cardiometabolic risk factors among 382,275 unrelated individuals of Europeans in the UK Biobank.
